# Specific Pathogen Recognition by Multiple Innate Immune Sensors in an Invertebrate

**DOI:** 10.3389/fimmu.2017.01249

**Published:** 2017-10-05

**Authors:** Guillaume Tetreau, Silvain Pinaud, Anaïs Portet, Richard Galinier, Benjamin Gourbal, David Duval

**Affiliations:** ^1^University of Perpignan, IHPE UMR 5244, CNRS, IFREMER, University of Montpellier, Perpignan, France

**Keywords:** invertebrate innate immunity, interactome, pathogen sensing, *Biomphalaria glabrata*, pattern-recognition receptor, proteomic profiling, immune specificity, hemocyte-free hemolymph

## Abstract

Detection of pathogens by all living organisms is the primary step needed to implement a coherent and efficient immune response. This implies a mediation by different soluble and/or membrane-anchored proteins related to innate immune receptors called PRRs (pattern-recognition receptors) to trigger immune signaling pathways. In most invertebrates, their roles have been inferred by analogy to those already characterized in vertebrate homologs. Despite the induction of their gene expression upon challenge and the presence of structural domains associated with the detection of pathogen-associated molecular patterns in their sequence, their exact role in the induction of immune response and their binding capacity still remain to be demonstrated. To this purpose, we developed a fast interactome approach, usable on any host–pathogen couple, to identify soluble proteins capable of directly or indirectly detecting the presence of pathogens. To investigate the molecular basis of immune recognition specificity, different pathogens (Gram-positive bacterium, *Micrococcus luteus*; Gram-negative, *Escherichia coli*; yeast, *Saccharomyces cerevisiae*; and metazoan parasites, *Echinostoma caproni* or *Schistosoma mansoni*) were exposed to hemocyte-free hemolymph from the gastropod *Biomphalaria glabrata*. Twenty-three different proteins bound to pathogens were identified and grouped into three different categories based on their primary function. Each pathogen was recognized by a specific but overlapping set of circulating proteins in mollusk’s hemolymph. While known PRRs such as C-type lectins were identified, other proteins not known to be primarily involved in pathogen recognition were found, including actin, tubulin, collagen, and hemoglobin. Confocal microscopy and specific fluorescent labeling revealed that extracellular actin present in snail hemolymph was able to bind to yeasts and induce their clotting, a preliminary step for their elimination by the snail immune system. Aerolysin-like proteins (named biomphalysins) were the only ones involved in the recognition of all the five pathogens tested, suggesting a sentinel role of these horizontally acquired toxins. These findings highlight the diversity and complexity of a highly specific innate immune sensing system. It paves the way for the use of such approach on a wide range of host–pathogen systems to provide new insights into the specificity and diversity of immune recognition by innate immune systems.

## Introduction

The innate immune system allows the host to sense pathogens and mount an appropriate anti-pathogenic defense. Confronted with a large variety of pathogens, ranging from viruses to multicellular parasites, the animals’ immune systems did not converge to a unique system with shared features but they emerged independently to provide an optimal protection of the host from infection (1). However, they all tend toward the genesis of a restricted repertoire of pathogen recognition molecules, named pattern-recognition receptors (PRRs), allowing to identify a determined diversity of pathogens (2). In vertebrates, pathogens recognition ability can be complemented by somatic recombination and hypermutation of a large repertoire of genes encoding immune receptors that lead to the production of soluble or membrane-bound antibodies (3, 4). Twelve years ago, Hargreaves and Medzhitov described the innate immune system in vertebrates as a complex of several recognition molecules capable of triggering one or more pathways to eliminate a given pathogen (1). Concepts highlighting the cooperation and complementation between the different recognition molecules leading to the activation of immune responses have since been supported by functional studies in vertebrates and in some model species (5, 6).

In invertebrates, and despite the lack of a vertebrate-like adaptive immunity, an increasing number of studies reported different repertoires of surprisingly highly diversified immune receptors within the innate immune system. This molecular diversity appears to be an essential basis for developing a fine and specific immune response against a large range of pathogens (7). The diversified arthropods’ Down syndrome cell adhesion molecule (Dscam) generated by different splicing events, the somatic hypermutated snail fibrinogen-related proteins (FREPs), the C-type lectins, or the sea urchin 185/333 proteins whose diversity is generated by RNA editing and post-translational modifications are the most well-known diversified immune molecules (8–10). However, they are not the only critical factors involved in pathogen recognition since their knock-out by RNA interference did not result in a complete lack of protection (11, 12).

Many additional actors have been characterized with the increasing use of high-throughput sequencing. Their annotation as “immune-like receptors” was based on the induction of their gene expression following infectious challenges and/or on the presence in their gene sequence of homologous domains already characterized in known immune receptors. Indeed, most immunological processes in invertebrates are extrapolated based on protein sequence homology with other model species (13–15). Moreover, many transcriptomic experiments performed in invertebrates following challenges with different pathogens resulted in a list of differentially expressed immune genes, supposedly involved in pathogen recognition, for which the interaction with pathogens and the potential roles in immune recognition have never been validated (16–18). As a consequence, many molecular functions still remain to be clarified, particularly their real contribution in the effective host immune response and the nature of the pathogen and/or molecular target with whom they interact.

To solve these questions, we investigated the immune sensing ability for a wide range of pathogens, from bacteria to trematodes, by the schistosomiasis vector snail, *Biomphalaria glabrata*. The objective of this study was to identify which molecules from the snail host interacted with pathogen’s surface determinants and their potential role in the specificity of the innate immune system. In this study, we report the repertoire of sensors from innate immunity constituted of previously characterized immune recognition factors (IRF) and of proteins involved in non-canonical immune pathways. These diverse and complementary molecules display a sentinel role by their constitutive expression in naïve animals. This circulating activity brings clues about the specificity and the mechanisms of pathogen detection in the host plasma. These results provide insights into the evolutionary selection of such factors and their role in specificity of invertebrate innate immunity that ultimately trigger an appropriate immune response, from inflammation to targeted clearance mechanisms.

## Materials and Methods

### Snail Rearing

An albino strain of the freshwater snail *B. glabrata* originated from Recife, Brazil (BgBRE2) was used as the invertebrate host (19). The snail strain was maintained in rearing chambers at 26°C, 12/12 h light/dark period. The laboratory and experimenters possessed an official certificate from the French Ministry of National Education, Research, and Technology, CNRS and DRAAF Languedoc Roussillon for experiments on animals, animal housing, and animal breeding (# A66040; decree # 87–848, October 19, 1987; and authorization # 007083).

### Hemolymph Extraction and Interaction with Pathogens

**Figure 1:**
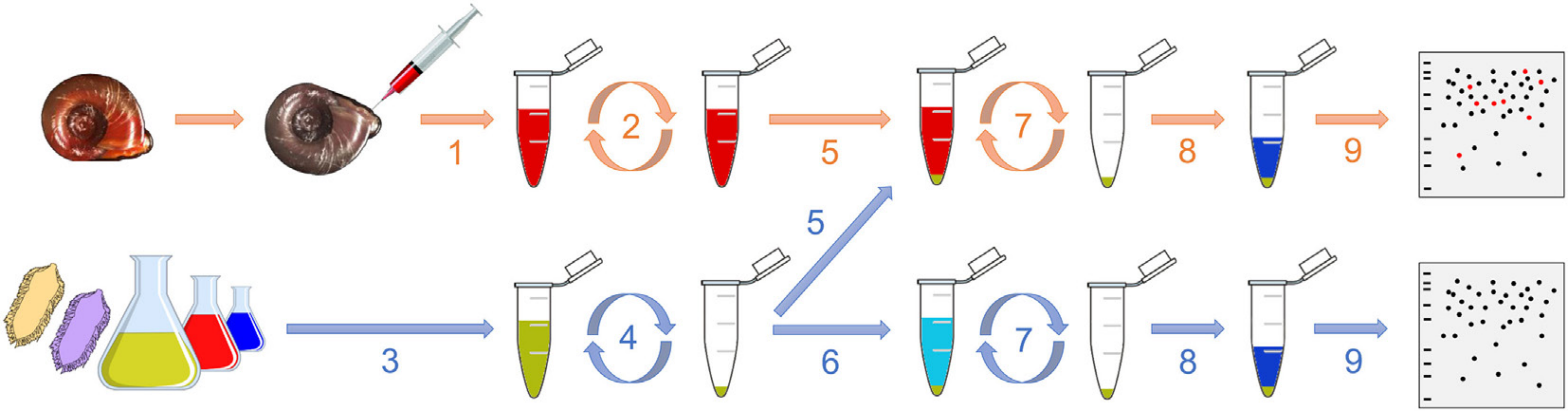
Graphical representation of the interactome procedure. The hemolymph is collected (1) and then centrifuged (2). Meanwhile, the pathogen is also collected (3) and centrifuged (4). The cell-free hemolymph is put in contact with the pellet of pathogen (5; “pathogens + hemolymph”). A control is also performed consisting in adding a buffer that mimics the internal snail osmolarity to the pellet of pathogen (6; “pathogens only”). After 20 min, the suspension is centrifuged (7), the pellet is washed and proteins are extracted (8) for their proteomic profiling by 2D-PAGE (9).

**Figure 2:**
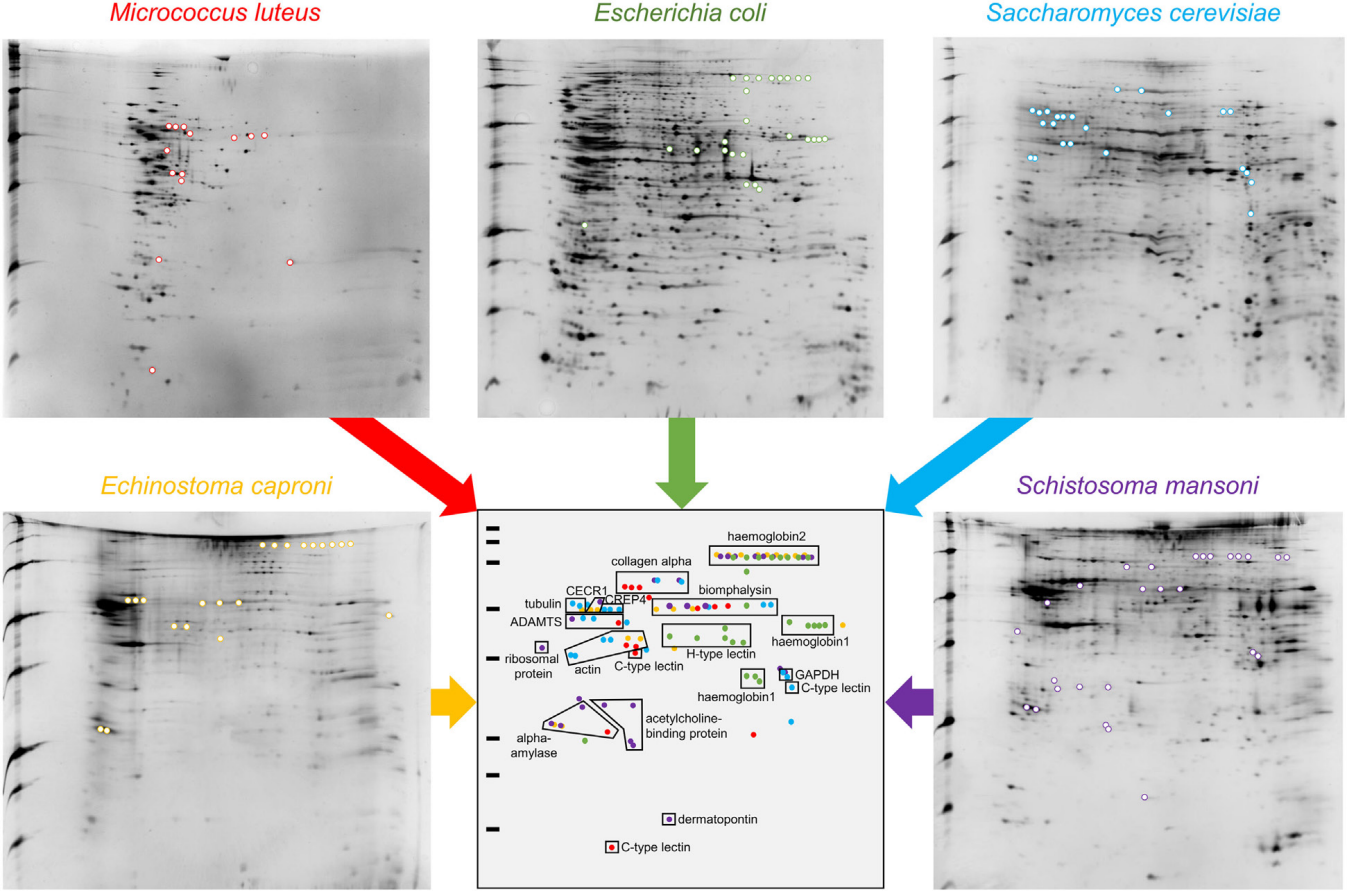
2D-PAGE gels of “pathogens + hemolymph” conditions. Colored spots are exclusively present in the “pathogens + hemolymph” profiles but not in the proteomic profiles of “pathogens only” (shown in Figure S1 in Supplementary Material). A schematic synthetic representation of the distribution of the spots exclusively present in the “pathogens + hemolymph” conditions is presented. Spots corresponding to proteins that interacted with the Gram-positive bacteria *Micrococcus luteus* are represented in red, those with the Gram-negative bacteria *Escherichia coli* in green, with the yeast *Saccharomyces cerevisiae* in blue, and with the trematodes *Echinostoma caproni* in orange and *Schistosoma mansoni* in purple.

The interactome procedure used in this study consists in comparing the proteomic profile of the pathogen alone with the proteomic profile of the pathogen that was in contact with the cell-free hemolymph from the snail (Figure 1). This allows identifying the native proteins from the hemolymph that interact with outer proteins from the entire living pathogen. Hemolymph was collected from the head–foot region of twenty 9- to 10-mm snails (Figure 1, 1) as previously described (20). 5 and 2 mL of hemolymph from a pool of snails were used for each replicate for interactome with bacteria and yeast and with metazoan parasites, respectively. Hemolymph was centrifuged at 2,000 × *g* for 10 min and the supernatant, constituting the cell-free hemolymph, was recovered for further interaction (Figure 1, 2). All plasma preparations were used immediately after their collection.

Integrity of the cells was verified by confocal microscopy to ensure that the procedure was not damaging the hemocytes, which could bias downstream analyses. Three conditions were tested: 1.freshly collected hemocytes were centrifuged at 2,000 × *g* for 10 min and used as a control for intact cells; 2. hemocytes vortexed and centrifuged at 2,000 × *g* for 10 min corresponded to the hemolymph preparation procedure of the interactome; 3. hemolymph sonicated (70% for 5 s) and then centrifuged (2,000 × *g* for 10 min) was the control of disrupted cells. Hemolymphatic cells were deposited on microscope slides to check their integrity and adhesion to surface. Cells were labeled with DAPI, which labels the DNA, and phalloidin, which labels the actin, by incubation for 20 and 2 min at 26°C in dark, respectively. Preparation was observed under a Zeiss LSM 700 microscope with two lasers at wavelengths of 405 and 488 nm for detection of DAPI and phalloidin labeling, respectively.

Five pathogens from three different kingdoms were used: the Gram-positive bacteria *Micrococcus luteus*, the Gram-negative bacteria *Escherichia coli*, the yeast *Saccharomyces cerevisiae*, and the two parasitic trematodes *Echinostoma caproni* and *Schistosoma mansoni*. *S. mansoni* and *E. caproni* have been maintained in the laboratory on *B. glabrata* BgBRE2 snails as previously described (12, 21).

**Figure 3:**
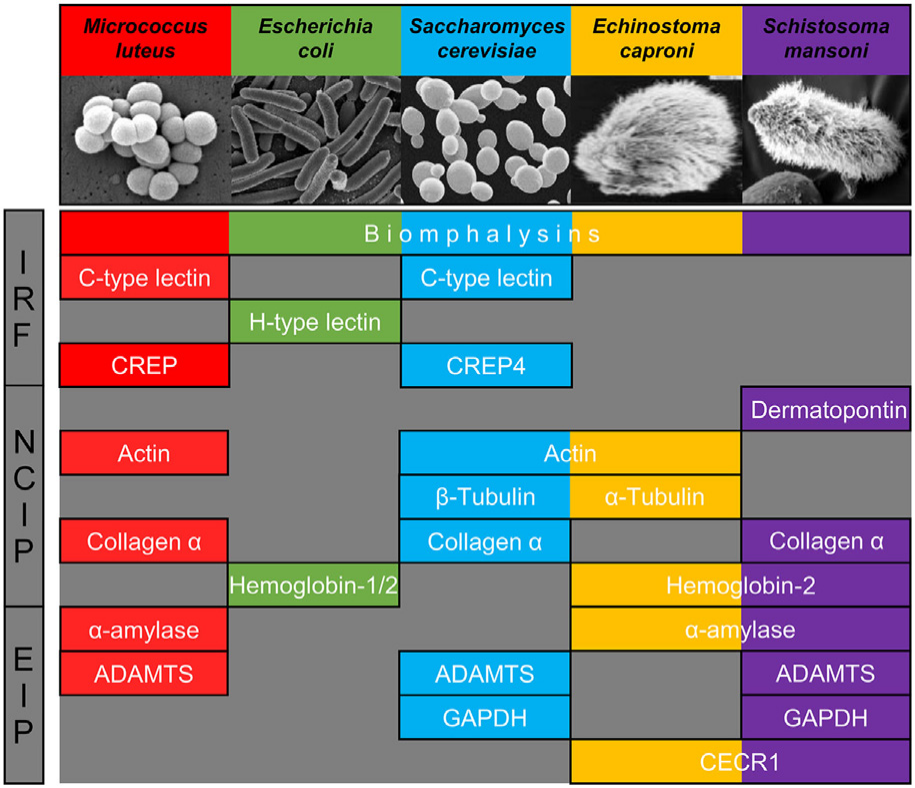
Major families of proteins implicated in recognition of at least one of the five pathogens used. They are classified in three categories: immune recognition factors (IRF), non-canonical proteins interacting with pathogens (NCIP), and enzymes interacting with pathogens (EIP).

**Figure 4:**
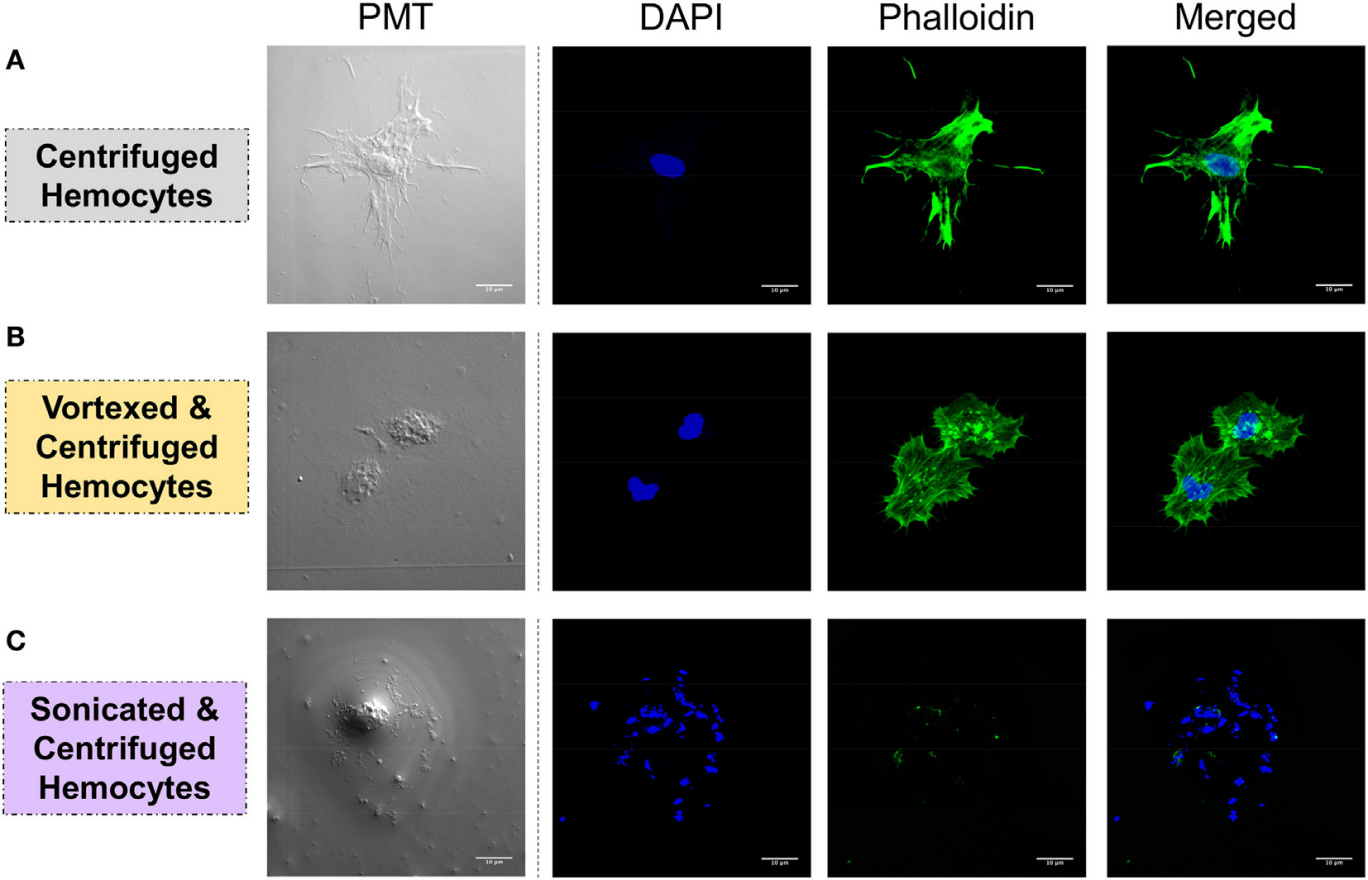
Hemocyte integrity was tested by analyzing the spreading capacity and by observing the nuclear/cytoplasmic ratio. Hemolymph was carefully collected and was either **(A)** slowly centrifuged, **(B)** vortexed and centrifuged, or **(C)** sonicated and centrifuged. Hemocytes were stained with DAPI, which colors nucleicacids contained in the nucleus in blue, and with phalloidin, which colors f-actin in green. White bar = 10 μm.

**Figure 5:**
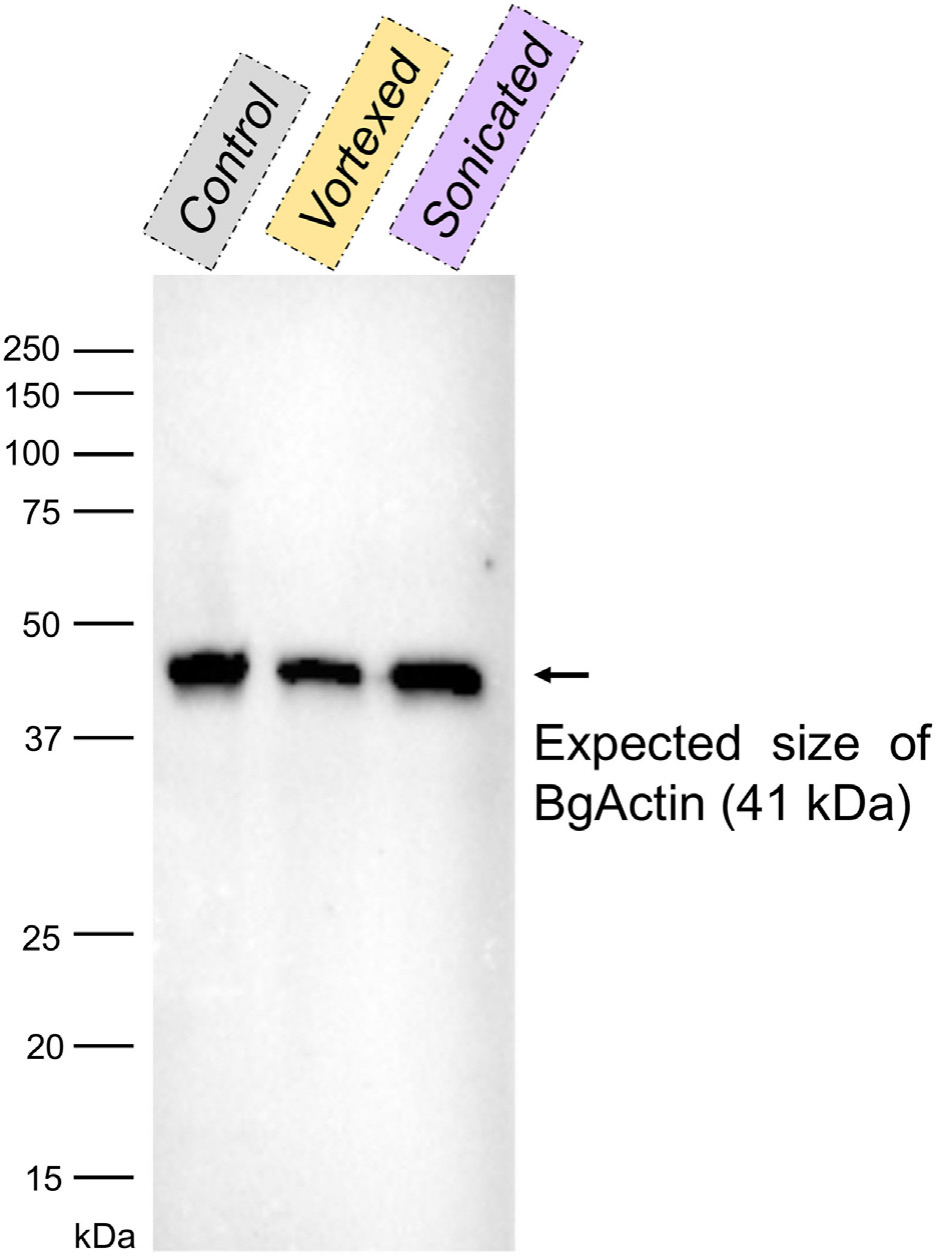
Western blot with anti-actin antibodies of the cell-free hemolymphs prepared by slow centrifugation (“control”), vortexing and centrifugation (“vortexed”) or sonication and centrifugation (“sonicated”). The band corresponding to the size of actin from *Biomphalaria glabrata* (~41 kDa; BgActin) is indicated by an arrow.

**Figure 6:**
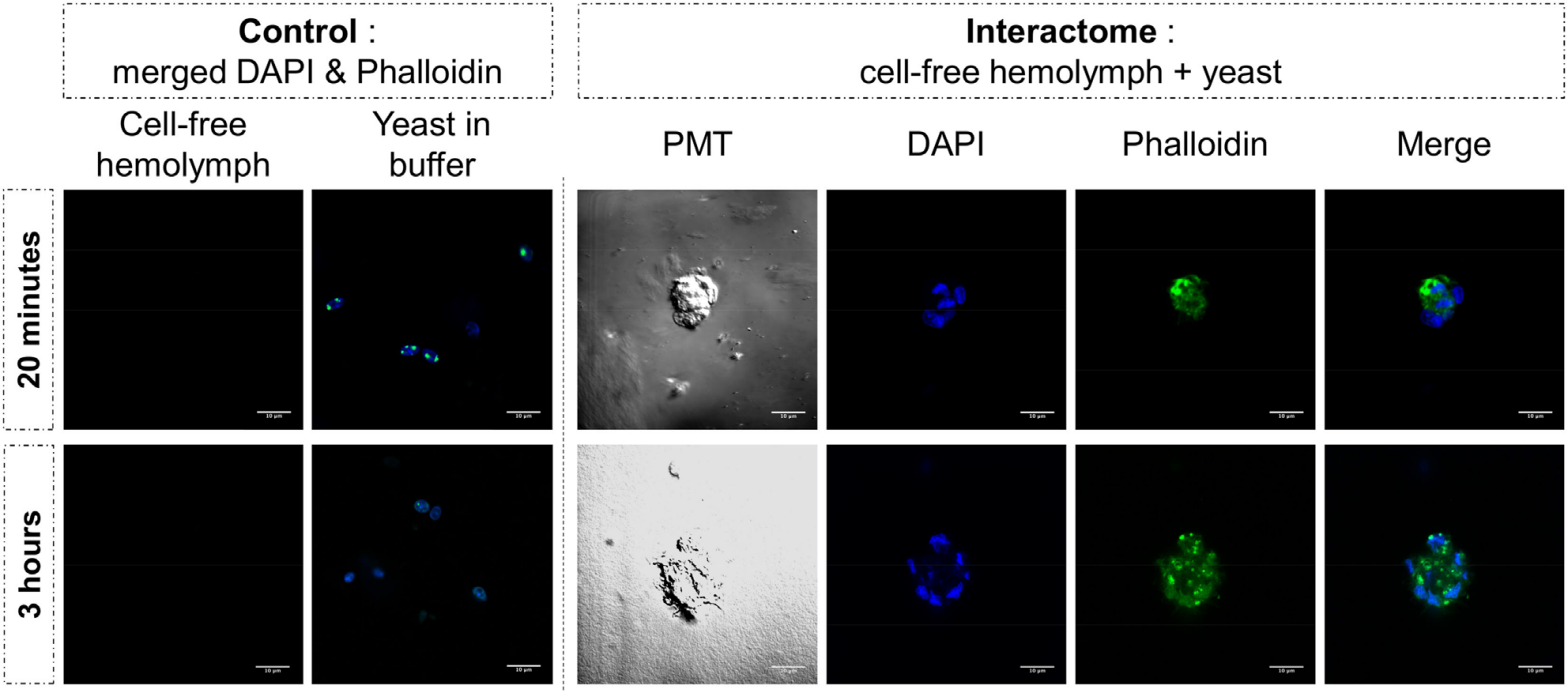
Yeast and cell-free hemolymph were used for an *in vitro* interactome. Actin was monitored by phalloidin labeling (in green) while nuclear compartment was revealed by acid nucleic labeling with DAPI (in blue). Photomultiplier tube (PMT) allowed visualizing cell membrane delimitation using white-light phase-contrast analysis. White bar scale = 10 μm.

The bacteria were plated and isolated on LB-agar Petri dishes. For each bacterium, one colony was introduced into a LB liquid medium and cultured overnight. Then, 150 μL of culture media, which contained approximately 35 million of bacteria, was sampled (Figure 1, 3) and centrifuged at 5,000 × *g* for 10 min (Figure 1, 4). This quantity of bacterial cells was based on studies previously published (22, 23) and it was shown to be above the detection threshold of the 2D-SDS-PAGE approach by preliminary tests (data not shown), which ensured a proper analysis of the interactome profiles. The supernatant was discarded and the pellet was washed twice with 1 mL of Chernin’s balanced salt solution (CBSS); NaCl, 48 mM; KCl, 2 mM; Na_2_HPO_4_, 0.5 mM; MgSO_4_⋅7H_2_O, 1.8 mM; CaCl_2_⋅2H_2_O, 3.6 mM; NaHCO_3_, 0.6 mM; pH 7.4. This buffer was chosen to mimic the internal snail osmolarity (24). The pellet was then resuspended in 1 mL of cell-free hemolymph and incubated on a rotating agitator for 20 min at 26°C (snail rearing chamber temperature) (Figure 1, 5). As a control, the bacterial pellet was incubated with 1 mL of filtered-CBSS in the same conditions (Figure 1, 6). After the incubation, the suspension was centrifuged at 5,000 × *g* for 10 min and the pellet was washed twice with 1 mL of CBSS (Figure 1, 7). Three biological replicates of each condition (“pathogen alone” and “pathogen + hemolymph”) were performed.

The yeast culture was performed on a unique colony in Sabouraud liquid medium (dextrose, 20 g L^−1^; pancreatic digest of casein, 5 g L^−1^; peptic digest of animal tissue, 5 g L^−1^, pH 5.6) at 26°C for 4 days. One hundred microliters of culture media, which contained approximately 30 million yeast cells, was collected as described above for bacteria.

*Schistosoma mansoni* eggs were recovered as previously described (12), then exposed to water and light for 2 h to let miracidia hatch. *E. caproni* adults were recovered on the digestive tracts of mice, cultured *in vitro* in RPMI solution supplemented with penicillin and streptomycin (SP4458, Sigma) at 37°C for 2 days. Eggs were recovered, washed, and stored in water in the dark at 26°C with air injector. Twenty days later, eggs were put in fresh water and exposed to light for 2 h for miracidia hatching. One thousand five hundred miracidia from *S. mansoni* and *E. caproni* were individually counted by using a glass pipette and processed as described for bacteria until protein extraction.

### Protein Extraction and 2D-SDS-PAGE Profiling

Proteins were extracted by resuspending the pellet of CBSS-washed pathogens in 70 μL of denaturing UTTC buffer (urea, 7 M; thiourea, 2 M; Tris, 30 mM; CHAPS, 4%; pH 8.5) (Figure 1, 8). After 2 h incubation at room temperature on a rocking agitator, the sample was centrifuged at 10,000 × *g* for 5 min and the supernatant was transferred to a low protein binding tube for its analysis by 2D-electrophoresis (Figure 1, 9).

Then, 280 μL of rehydration buffer (urea, 7 M; thiourea, 2 M; CHAPS, 4%; DTT, 65 mM) containing 0.2% of Bio-Lyte 3/10 ampholyte (Bio-Rad) was added. The sample was then loaded on a tray channel for 5 h of passive rehydration followed by 14 h of active rehydration (50 V) of a 17 cm ReadyStrip IPG strip with a non-linear 3–10 pH gradient (Bio-Rad). Focusing was performed using the following program: 50 V for 1 h, 250 V for 1 h, 8,000 V for 1 h, and a final step at 8,000 V for a total of 90,000 V h with a slow ramping voltage (quadratically increasing voltage) at each step. Rehydration and focusing were both performed on a Protean IEF Cell system (Bio-Rad). Focused proteins were reduced by incubating the strip twice with equilibration buffer (Tris, 1.5 M; urea, 6 M; SDS, 2%; glycerol, 30%; bromophenol blue; pH 8.8) containing DTT (130 mM) at 55°C and they were alkylated by an incubation with equilibration buffer containing iodoacetamide (135 mM) on a rocking agitator (400 rpm) at room temperature protected from light.

Proteins were separated in function of their molecular weight on a 12%/0.32% acrylamide/piperazine diacrylamide gel run at 25 mA/gel for 30 min followed by 75 mA/gel for 8 h using a Protean II XL system (Bio-Rad). Protein standards were loaded with Whatman paper impregnated with 3 μL of Unstained Precision Plus Protein Standards (Bio-Rad) on the left part of the gels. Gels were stained following a regular silver staining procedure: sensitizing using sodium acetate (68 g L^−1^) and sodium thiosulfate (2 g L^−1^), marking with 2.5 g L^−1^ of silver nitrate, and then developing with sodium carbonate (25 g L^−1^) in a 7.5% formaldehyde solution. Staining was stopped by replacing the developing solution by a solution of glycine (5 g L^−1^) in 0.1% acetic acid. Gels were scanned using a ChemiDoc MP Imaging System (Bio-Rad) associated with Image Lab software version 4.0.1 (Bio-Rad). The qualitative comparative analysis of digitized proteome maps was conducted using the image analysis software PDQuest 7.4.0 (Bio-Rad). Only spots present in all the three replicates of “pathogens + hemolymph” samples and absent from all the profiles of pathogens alone were selected and picked in a mass spectrometry (MS)-compatible silver stained gel for further identification.

### Spot Picking and Trypsin Digestion

Spots were excised from the gels using a Onetouch Plus Spot Picker Disposable (Harvard Apparatus), equipped with specific 1.5-mm methanol-washed tips. The gel plug containing the spot was disposed into a methanol-washed low protein binding tube and stored at −80°C until further processing. Gel plug was first destained by incubating it in 150 μL of a solution of potassium ferricyanide (15 mM) and sodium thiosulfate (50 mM) at room temperature for 10 min on a rocking agitator (500 rpm). The destaining solution was discarded and this step was repeated once. Then, the plug was washed twice by adding 150 μL of ammonium bicarbonate (25 mM) and it was incubated at room temperature for 30 min on a rocking agitator (500 rpm). Finally, 150 μL of a solution of ammonium bicarbonate (12.5 mM) and acetonitrile (50%) was added to the spot. After incubation at room temperature for 10 min on a rocking agitator (500 rpm), the solution was discarded and the gel plug lyophilized for 30 min. The plug was rehydrated with 50 μL of sequencing grade modified trypsin (Promega) and incubated on ice for 30 min. The excess of trypsin was discarded and 50 μL of ammonium bicarbonate (25 mM) was added. Digestion was performed overnight at 30°C. The 50 μL of solution were put in a new methanol-washed low-protein binding tube and the peptides were extracted from the plug by washing it three times with 100 μL of a solution of formic acid (1%) and acetonitrile (50%) and by incubating 15 min at room temperature on a rocking agitator (500 rpm). The solution was collected at each washing step and mixed together in the same tube (final volume: 350 μL). The solution was flash-frozen in liquid nitrogen, lyophilized for 3 h and stored at −80°C until further processing.

### MS/MS Identification

Peptides were resuspended in 10 μL of 3% (v/v) acetonitrile and 0.1% (v/v) formic acid, and then analyzed with a nano-LC1200 system coupled to a Q-TOF 6550 mass spectrometer equipped with a nanospray source and an HPLC-chip cube interface (Agilent Technologies). A 34-min linear gradient (3–75% acetonitrile in 0.1% formic acid), at a flow rate of 350 nL min^−1^, was used to separate peptides on a polaris-HR-Chip C18 column (150 mm long × 75 μm inner diameter). Full autoMS1 scans from 290 to 1700 *m/z* and autoMS2 from 59 to 1700 *m/z* were recorded. In every cycle, a maximum of five precursors sort by charge state (2+ preferred and single-charged ions excluded) were isolated and fragmented in the collision cell that was automatically adjusted depending on the *m/z*. Active exclusion of these precursors was enabled after 1 spectrum within 0.2 min, and the absolute threshold for precursor selection was set to 1,000 counts (relative threshold 0.001%). For protein identification, peak lists were extracted (merge MSn scans with the same precursor at ±30 s retention time window and ±50 ppm mass tolerance) and compared with specific databases by using the PEAKS studio 7.5 proteomics workbench (Bioinformatics Solutions Inc., build 20150615). The searches were performed with the following specific parameters: enzyme specificity, trypsin; three missed cleavages permitted; fixed modification, carbamidomethylation (C); variable modifications, oxidation (M), pyro-glu from E and Q; monoisotopic; mass tolerance for precursor ions, 20 ppm; mass tolerance for fragment ions, 50 ppm; MS scan mode, quadrupole; and MS/MS scan mode, time of flight. For each interactome experiment, each spot identification was performed against the *B. glabrata* translated transcriptome (12, 25) and against the corresponding pathogen proteome. Only significant hits with a false discovery rate (FDR ≤ 1) for peptide and protein cutoff (−logP ≥ 20 and number of unique peptides ≥2) were considered. For ensuring a proper identification of the proteins found by the interactome approach, a BLAST search against NCBI nr database was performed and the conserved domains of the sequence were retrieved using the NCBI CD-search available at https://www.ncbi.nlm.nih.gov/Structure/cdd/wrpsb.cgi (26). For each protein, pI and molecular mass were also calculated with the ExPASy Compute pI/Mw tool (available at http://web.expasy.org/compute_pi) to compare with their location on the gel and provide an additional confirmation of their proper identification.

### Validation of Actin As an Extracellular Immune Factor

Integrity of the cells was verified by confocal microscopy prior to actin localization in the plasma to ensure that the preparative procedure was not damaging the hemocytes, which could bias downstream analyses. The same three samples of hemolymph used for cell integrity (centrifuged hemolymph, vortexed and centrifuged hemolymph, and sonicated and centrifuged hemolymph) were used. 40 μL of hemolymph from each sample were extracted in Laemmli buffer (Bio-Rad) containing β-mercaptoethanol and denaturated at 99°C for 5 min. Proteins were separated in a 12% acrylamide gel using the Mini-Protean Tetra Cell machinery (Bio-Rad) powered by PowerPac HC (Bio-Rad) at 110 V for 80 min. Proteins were then transferred onto a 0.2 μm PVDF membrane using Trans-Blot Turbo Transfer Pack for 3 min at 25 V and 2.5 A (Bio-Rad). After saturation during 1 h at 37°C in TBSTM [1× TBS (500 mM Tris-HCl, 1.5 M NaCl, pH 7.5), 0.05% Tween20, 5% non-fat milk], the membrane was incubated for 90 min at RT in TBSTM containing a mouse actin monoclonal antibody (mAbGEa, ThermoFisher) at a 1:1,000 dilution. The membrane was washed three times with TBST (TBSTM without milk), and further incubated for 70 min at RT with manufactured horseradish peroxidase-conjugated goat anti-mouse IgG antibody (Agrisera) at a 1:4,000 dilution. The membrane was washed three times with TBST. Actin presence was revealed by incubating the membrane in an enhanced chemiluminescent reagent (Super Signal West Pico Chemiluminescent Substrate, ThermoScientist) for 5 min at RT. The membrane was scanned using a ChemiDoc MP Imaging System (Bio-Rad) associated with Image Lab software version 4.0.1 (Bio-Rad).

### Yeast Clotting by Incubation with Cell-Free Hemolymph

Yeast cells were cultured in Sabouraud medium as described above. They were washed twice with CBSS. Yeasts were then resuspended either in CBSS or in cell-free hemolymph for 20 min or 3 h. Preparations were deposited on microscope slides for platting and were then labeled with DAPI and phalloidin as described above. They were observed using a Zeiss LSM 700 microscope.

## Results and Discussion

### An Original and Simple Method

Generally, the identification of host molecules that can bind or recognize a set of pathogen determinants is performed by global pull-down assays. Such global interactome approach consists in the incubation of native or denatured protein extracts from both the host and the pathogen. The resulting interacting protein complexes are then separated through differential centrifugation steps, revealed by SDS-PAGE and identified by MS (27–29). Although powerful, this strategy suffers from several flaws, mainly associated with the extraction procedure itself which might (i) affect the nature of protein interactions by changing their conformation and (ii) promote forced interaction between proteins that would not encounter each other *in vivo*. Therefore, a part of the interactions observed can be essentially artificial and experimentally biased. To bypass these problems, we propose a new and simple interactome procedure in a cell-free hemolymph context that tends to mimic biological interactions between pathogens and soluble host proteins (Figure 1). Indeed, entire living pathogens were exposed to circulating humoral factors already present in cell-free hemolymph freshly extracted from naïve snails and they were incubated at 26°C, which corresponds to the environmental and internal temperature of this ectothermic organism. Therefore, only proteins present at the surface of the pathogen are recognized in a biologically realistic context. Moreover, the short time (20 min) chosen allows focusing exclusively on the very first step of innate immune response and avoiding the pathogen to respond to the attack from the immune factors, which could affect pathogens’ proteomic profiles and bias the analysis. As a control, only spots that were present in the three “pathogen + hemolymph” replicates and absent in the three “pathogen only” replicates were considered for the analysis of each pathogen studied. Each MS/MS profile was confronted to both the databases of the host and of the pathogen. This ensured that the approach reliably enabled the identification of host’s interacting molecules while limiting the risk of false positives. No significant matches were observed against any of the pathogen databases, which confirms that all spots exclusively identified in the analysis of “pathogen + hemolymph” samples and not in the “pathogens only” gels were proteins from the snail’s plasma. The benefit of this approach relies on its universality: it can be used with most host and parasite systems and gives rise to reliable qualitative differences within just few hours, which represents a great step forward for studies focusing on model and non-model systems.

### Identification of a Large Variety of Interacting Proteins

This approach allowed the identification of a total of 109 spots exclusively identified in “pathogen + hemolymph” samples for the five pathogens tested (Figure 2; Figure S1 in Supplementary Material). These spots provided a significant match to 34 unique accession numbers, referring to 23 different proteins (Table S1 in Supplementary Material). Each pathogen was recognized by a specific, but overlapping, set of circulating proteins in mollusk’s hemolymph (Figure 3). Specific recognition proteins to a given pathogen must be expected since each class of pathogen express at their surface specific and different structural motif also called pathogen-associated molecular patterns (PAMPs). The best known PAMPs are lipopolysaccharide from Gram-negative bacteria, lipoteichoic acid or peptidoglycan from Gram-positive bacteria, mannan-derived molecules or glycan from fungi, and fucosylated or glycoprotein receptors from *Schistosoma* sp. (30). Surprisingly, we identified numerous proteins not known to be involved in pathogen recognition and/or killing (extracellular matrix proteins, protease, and carbohydrase enzyme). Considering that some of these proteins are generally considered as intracellular molecules, a possible explanation for their presence could be that host’s cells were damaged during the hemolymph collection (although non-invasive) and/or during the centrifugation step. A dual-staining with DAPI and phalloidin of hemocytes revealed no difference between fresh hemocytes and vortexed ones that were intact, as compared to sonicated hemocytes that were totally disrupted (Figure 4). This confirmed that the procedure of preparation of cell-free hemolymph did not damage the cells and that all interacting proteins from the snails were naturally present in the extracellular compartment of the hemolymph.

We, thus, propose to classify the snail interacting proteins identified into three different categories based on their nomenclature and known primary function: (i) molecules previously described as primary pathogen recognition molecules able to trigger an immunological response, with potential additional lytic activity [immune recognition factors (IRF)], (ii) proteins whose primary role is not pathogen sensing but are involved in other physiological functions [non-canonical proteins interacting with pathogens (NCIP)], and (iii) enzymes implicated in the metabolism of a wide range of molecules enzymes interacting with pathogens (EIP).

### Pathogen Sensing by Soluble Immune Receptors and Atypical Toxins (IRF)

Among the IRF, two different families of proteins are identified: lectins and biomphalysin (Figure 2). Lectins represent a large family with a wide variety of evolutionarily conserved structures and some of them have been described as involved in immune recognition (7, 31). Among them, calcium-dependent (C-type) lectins were considered the most promising pattern-recognition proteins involved in the specific recognition of pathogens in the invertebrate immune system. This specificity is due to their high level of polymorphism and/or diversification to face up pathogens’ antigenic diversity (31). In addition to their role as soluble receptors, they can also limit the spreading of the pathogen in the host’s tissues and participate to its elimination (32, 33). Two different C-type lectins were interacting with the bacterium *M. luteus* and the yeast *S. cerevisiae* but not with the three other pathogens (Figure 2; Table S1 in Supplementary Material). Another C-type lectin-related protein (CREP4), recently characterized in *B. glabrata* from transcriptomic data (25), was apparently able to bind to *S. cerevisiae*. By contrast, the recognition of the bacterium *E. coli* involved a totally different category of lectin, the hyal-adherins (H-type), which are also carbohydrate-binding proteins but data are missing concerning their role in pathogen recognition. Among the lectins, FREPs are proteins containing immunoglobulin-like domains whose role in the interaction between snails and metazoan parasites has been suggested (34, 35). Surprisingly, FREPs were not identified in the interaction with both metazoan parasites in our study while they were evidenced in previous transcriptomic and proteomic studies (27, 29). Such discrepancy with previous results likely comes from the different developmental stage of the parasites used in the different studies, i.e., miracidia herein and sporocysts in other studies. Several proteomic and glycomic studies showed that the glycan elements harbored by *Schistosoma*, to which FREPs bind, differ from one developmental stage to another (36, 37). This would suggest a subtle ability for the snail immune machinery to distinguish various intramolluscal developmental stages of the parasite (miracidium to primary and secondary sporocysts or even cercariae) and FREPs might not be involved in the recognition of all stages. Moreover, FREPs were previously identified by interactome experiments after 2.5 h of contact between protein extracts from sporocyst and snail cell-free hemolymph (27) while our procedure includes a 20-min contact of outer pathogen membrane proteins with circulating snail hemolymph proteins. Of note, it has been observed that some FREPs can form multimers and that they can interact with other proteins such as thioester-containing proteins (TEPs), which could both modulate their recognition ability (27, 34, 38). It is, therefore, possible that these processes are mandatory for the recognition by FREPs of the pathogens used in this study. A longer exposure time between pathogens with proper membrane-bound glycan antigens and the cell-free hemolymph would then be required for the complexes to form and for their detection by our interactome approach.

The second class of IRF identified is the biomphalysin toxin, which is an aerolysin-like protein that has been acquired by a putative horizontal gene transfer from a bacterium (39) (Figure 3). This protein is constituted of two domains: one large domain that shares structure similarities with β-pore-forming toxins whose role is to perforate cell membranes by forming transmembrane pores and a small domain potentially involved in pathogens’ carbohydrate motifs recognition (39). Biomphalysin is a dual protein: it has recently been shown to directly bind to *S. mansoni* sporocysts and to have a lytic activity enhanced by snail plasmatic factors (39). Herein, we demonstrate for the first time that this anti-schistosome toxin is also able to interact with other pathogens and suggest a role in bacterial clearance. One (*E. coli*) and three (*M. luteus, S. cerevisiae, E. caproni*, and *S. mansoni*) spots were identified as biomphalysins in 2D gels (Figure 2; Table S1 in Supplementary Material). Even if they were all of the same size (65–70 kDa), the expected size of biomphalysin (39), they exhibited a large range of isoelectric points, from slightly acid/neutral for *E. caproni* and *S. mansoni* to basic for *E. coli* and *S. cerevisiae* (Figure 2). Altogether, this suggests that different protein isoforms of biomphalysins must be involved in the recognition/clearance of the same pathogen but also of different pathogens. Interestingly, different biomphalysin genes were predicted in the recently sequenced genome of *B. glabrata* (BioProject: PRJNA290623 on NCBI database) (40), which suggests that they might be different genes rather than different isoforms (39). This biomphalysin family could be a major player of the specificity of the *Biomphalaria* innate immune response together with lectins.

Biomphalysins were the only proteins that interacted with all pathogens. There is a growing number of evidence that aerolysin-like proteins have been horizontally transferred within many different invertebrate phyla acquiring in the same time potentially new and varied functions but details of their involvement in the invertebrate immunity remain largely unknown (41). The interactome approach developed herein suggests that biomphalysins might be a key component of the pathogen sensing system, and potentially of its specificity. Indeed, heterogeneous assembly from these different monomeric isoforms to the heptameric biomphalysin pore complex may generate a high degree of pathogen-binding specificity. In *Anopheles gambiae*, two C-type lectins, CTL4 and CTLMA2, form a disulfide-linked heterodimer to specifically kill *E. coli* (42). The ability to form heterodimers could greatly expand the repertoire of recognition molecules (43, 44). Further experiments are now required to understand how biomphalysin gene expression is regulated in response to exposure with different pathogens and how the different proteins are recruited to respond to a specific pathogen encounter.

### Pathogen Sensing by Major Extracellular Matrix Components (NCIP)

The category of NCIP includes proteins whose primary function is not immunity, such as cell-matrix junction proteins (dermatopontin, collagen) and cytoskeleton extracellular matrix proteins (actin, tubulin). Concerning the dermapontin, its gene expression can be increased after immune challenge with *E. caproni* (21, 45) and *S. mansoni* (45) but not with *E. coli, B. cereus*, and *S. cerevisiae* (46). While its role was unknown at this time, our results suggest that it might be involved in a hemolymph coagulation-like system to prevent parasite establishment through the tissue of the host (Figure 3).

The same type of molecular process is expected for other extracellular proteins such as actin. Western blot analyses of cell-free hemolymph using anti-actin antibodies revealed its presence in the extracellular compartment of the hemolymph (Figure 5). Considering that the procedure of hemolymph collection and preparation did not damage the cells (Figure 4), this actin must be considered as a real extracellular actin (ECA) present in snail hemolymph. Interestingly, the amount of ECA present in hemolymph was similar between the three conditions tested in western blot, which suggests that ECA is an important component of hemolymph released by a process still unknown in mollusk. In insects, some isoforms are secreted from cells through an exosome-independent pathway (47) while monocyte cells can release some extracellular vesicles (ectosome) containing b-actin and actinin in vertebrates (48). Observation of yeasts by confocal microscopy shows that in CBSS buffer, some actin is located inside the yeast, revealed as small precisely localized green dots (Figure 6). In the presence of cell-free hemolymph, these intra-yeast dots of actin are still visible but there is a large amount of ECA surrounding the yeast cells, which appears as early as 20 min and seems even more intense after 3 h of incubation (Figure 6). Considering that yeasts were still intact after 20 min of contact with cell-free hemolymph, this actin surrounding the yeasts is likely the ECA from snail that is able to bind and participate to yeast clotting prior to its elimination. The triggering of the destruction of yeast cells by these immune complexes is indicated by their nuclear destructuration visible at 3 h (Figure 6). This finding is consistent with recent studies that demonstrated an active role of actin in extracellular trap for pathogens clotting, facilitating their elimination by phagocytosis in the mosquito *A. gambiae* for example (47). Until now, these soluble molecules were considered as damage-associated molecular patterns (DAMPs) potentially involved in the “danger theory” where self-constituents could trigger an immune response (49). Based on our results and particularly on the short time of our interaction that prevents the pathogen from circumventing host immune factors, these molecules must be considered as soluble immune sensing factors rather than just DAMPs.

The case of hemoglobin is particularly interesting. Two different classes of hemoglobin were identified against *E. coli* (hemoglobin-1 and -2) while only hemoglobin-2 was interacting with *E. caproni* and *S. mansoni* (Figure 2). Many different isoforms were identified (same size, different isoeletric points) but they were at a much lower size (55–60 and 100–120 kDa for hemoglobin-1 and -2, respectively) than the predicted full-size hemoglobin protein predicted from *B. glabrata* genome (514 and 582 kDa, respectively) (Figure 2). Such peptides with enhanced or alternative functionality that can be liberated from larger proteins are named cryptides. Those derived from hemoglobin have already been associated with immune modulation, hematopoiesis, signal transduction, and microbicidal activities in metazoans (50). Although identified as differentially expressed upon *S. mansoni* exposure in *B. glabrata* (45), these highly abundant proteins were excluded from previous interactome approaches by ultracentrifugation of plasma as they were thought to interfere with pathogen recognition and not be directly implicated in it (27). Also, the role of this major protein in hemolymph has been largely neglected as its function was expected to be mostly pleiotropic. Hemoglobin and/or hemoglobin cryptides could directly interfere with the pathogen and limit its growth, as it has been shown for the “classical swine fever virus” (51), and/or they could reinforce the interaction between pathogen and extracellular matrix proteins, as it has been shown between human fibronectin and the pathogenic yeast *Candida albicans* (52). The binding of hemoglobin to the major virulence factor of *Salmonella typhi* has also been shown to promote the production of proinflammatory cytokines from monocytes (53).

### Host Plasmatic Enzymes Involved in Pathogen Surface Binding (EIP)

Many different EIPs were identified in this interactome approach (Figure 3). α-amylases have already been identified after co-immunoprecipitation of *B. glabrata* plasmatic proteins with *S. mansoni* protein extracts but they were considered as mucus contamination at this time (27). Present data challenge this contamination hypothesis since α-amylase was only detected after interaction of hemolymph with *M. luteus*. α-amylases would, thus, be critical for the host’s specific response to certain pathogens. For the other EIPs, reports on the involvement of ADAMTS, GAPDH, and CECR1 in invertebrate immunity are scarce. However, GAPDH has been demonstrated to modulate immune responses against bacteria in plants (54) and metalloproteases have been characterized as key actors of many diverse immune and inflammatory processes in vertebrates (55). Results obtained in this study demonstrate that their binding to the pathogen surface can no longer be considered as artifactual. Further experiments are now required to understand if EIPs can bind directly to surface pathogens’ factors or if their involvement is related to their enzyme activities to mediate the maturation of immune complexes after association with other IRFs and/or NCIPs.

### Experimental Support to Theoretical Concepts Opens New Perspectives for Studying Pathogen Sensing by Invertebrates

Although extensively investigated and well documented in vertebrates, the factors involved in invertebrate immune recognition rather constitute a black-box in which many different proteins with a wide range of functions, often referred to as PRRs, can be found (56, 57). Some responses have arisen from model species essentially from insects such as *Drosophila* for which the Gram-negative bacteria-specific Imd pathway and the fungi and Gram-positive bacteria-specific Toll pathway have been first identified (8). However, data remain scarce in non-model species mostly due to the absence of reliable knock-out technology, which may fail in demonstrating the full richness and the role of invertebrate pathogen recognition molecules (7, 58).

In this study, we developed a simple interactome approach to identify soluble plasmatic molecules that bind directly or indirectly to pathogen surfaces and to gain access rapidly to the biological functions of the candidate proteins. Here, we focused on the sentinel role of molecules that interacted with pathogens since they were constitutively present in hemolymph of uninfected (naïve) snails. Indeed, most of the studies are based on the differential analysis (i.e., uninfected vs infected, or infected by different pathogens) of the host immune response (efficient or not) leading to a list of genes whose immunological function is rarely demonstrated. Moreover, if functional invalidation (gene knock-out, siRNA-mediated gene silencing, and mutants) already demonstrated the requirement of such molecules during the immune response, the first step of pathogens binding is still rarely studied (11, 12, 33).

Each pathogen was recognized by a specific, although partially overlapping set of interacting proteins from the mollusk (Figure 3; Table S1 in Supplementary Material). Most of pathogens’ perception involved at least three different families of proteins from two of the three protein categories described (Figure 3). Such contrasting sets of binding proteins, in terms of diversity and quantity, suggest that specificity of immune detection quickly occurs at a fine scale. The recognition of the same pathogen by several different sensors with a high degree of specificity suggests that these molecules are part of different host defense pathways that can interact with each other (1). Such interactions can take three different forms: by cooperation, leading to the more efficient engagement of the same effector mechanism, by complementation, allowing to trigger different complementing effector mechanisms or by compensation, where one pathway compensates the deficiency of another one (59). The real involvement of these proteins in pathogen recognition, as expected in parasite antigen/host receptor interaction, is still not demonstrated and will require specific investigation of downstream process for each candidate identified. Thus, these pathways might contribute to assess the danger for which they have been exposed and leading *in fine* to discriminate symbiotic organisms from pathogens (60). Simultaneous activation of distinct recognition pathways would enable a concerted and appropriate response to tolerate or eliminate such or such intruder. Another aspect of the molecular interaction not yet described and evaluated in invertebrates is the temporal dynamic of pathogen perception by soluble immune factors. Can this recognition be immediate and frozen once and for all, or require gradual biochemical and structural maturation to recruit other more specialized immune factors? The dynamic of structuration of soluble immune complexes by analyzing interactome at different times must be explored to answer this question. In this study, we show that different biochemical interactions between the external surface of pathogens and host molecules occur within just 20 min of interaction. This supports the idea of a first wave of pathogen detection that we called “sensing,” a prerequisite for the subsequent activation of immune system. This sensing step appears additive but also epistasic by the number of various biological functions involved and suggests a cooperative crosstalk for a specific immune response (1). The relative function of the IRF, NCIP, and EIP, whether they are implicated in pathogen recognition, immune complex maturation, and/or triggering of immune response, will require further investigation. The method developed herein allowed reaching the early step of pathogen sensing, validating the binding ability of several IRF, and opening opportunity in model systems to deeper study their activity in the immune response pathways.

In summary, the present data constituted an important step toward a better understanding of the pathogen sensing and immune specificity in invertebrates. It clearly demonstrates that innate immune response in invertebrate is not supported by a unique class of immune factors but rather by a panel of molecules involved in diverse biological functions and able to bind specifically to a range of distinct pathogens. Notably, it involves some dual immune proteins able to play a role in both pathogen binding and clearance. This work does not intend to provide an extensive description of all sensing molecules but it definitely opens the way to a better integrative biological overview of molecules necessary to initiate an orchestrated immune response against pathogens in both model and non-model organisms.

## Author Contributions

GT, SP, and DD designed the research; AP, RG, and BG substantially participated in conception and improvement of research; SP, AP, and DD performed interaction experiments; GT performed the 2D-SDS-PAGE experiments and qualitative analysis; SP and AP performed the Western blots, fluorescent labeling, and microscope observations; all authors contributed to the analysis and interpretation of the results; GT, SP, and DD led the manuscript writing; all authors participated to manuscript writing, editing, and critical reviewing; and they all approved the final draft.

## Conflict of Interest Statement

The authors declare that the research was conducted in the absence of any commercial or financial relationships that could be construed as a potential conflict of interest. The funders had no role in study design, data collection and interpretation, or the decision to submit the work for publication.

## Supplementary Material

Figure S1 2D-PAGE gels of “pathogens + hemolymph” and “pathogens only” for each of the five pathogens used. Arrows are indicating spots exclusively present in the “pathogens + hemolymph” profiles but not in the proteomic profiles of “pathogens only,” which represents proteins from *Biomphalaria glabrata* hemolymph that participated in the recognition of pathogen’s proteins.

Table S1 Protein identification of the 109 spots revealed only in “pathogens + hemolymph” gels as compared to “pathogens only” gels. For each spot, the −10logP values of proteins and peptides are indicated, together with the top BLAST hit in NCBI nr database, the conserved domains of the sequence retrieved (performed with NCBI CD-search available at https://www.ncbi.nlm.nih.gov/Structure/cdd/wrpsb.cgi) and the pI and molecular mass (calculated with the ExPASy “Compute pI/Mw tool” available at http://web.expasy.org/compute_pi).
