# Supplementary material for: Specific Pathogen Recognition by Multiple Innate Immune Sensors in an Invertebrate
Source: Front Immunol. 2017 Oct 5;8:1249. doi: 10.3389/fimmu.2017.01249 (PMC5633686; doi:10.3389/fimmu.2017.01249)
Supplement: Figure S1 — 2D-PAGE gels of “pathogens + hemolymph” and “pathogens only” for each of the five pathogens used. Arrows are indicating spots exclusively present in the “pathogens + hemolymph” profiles but not in the proteomic profiles of “pathogens only,” which represents proteins from Biomphalaria glabrata hemolymph that participated in the recognition of pathogen’s proteins. [file Image_1.PDF]

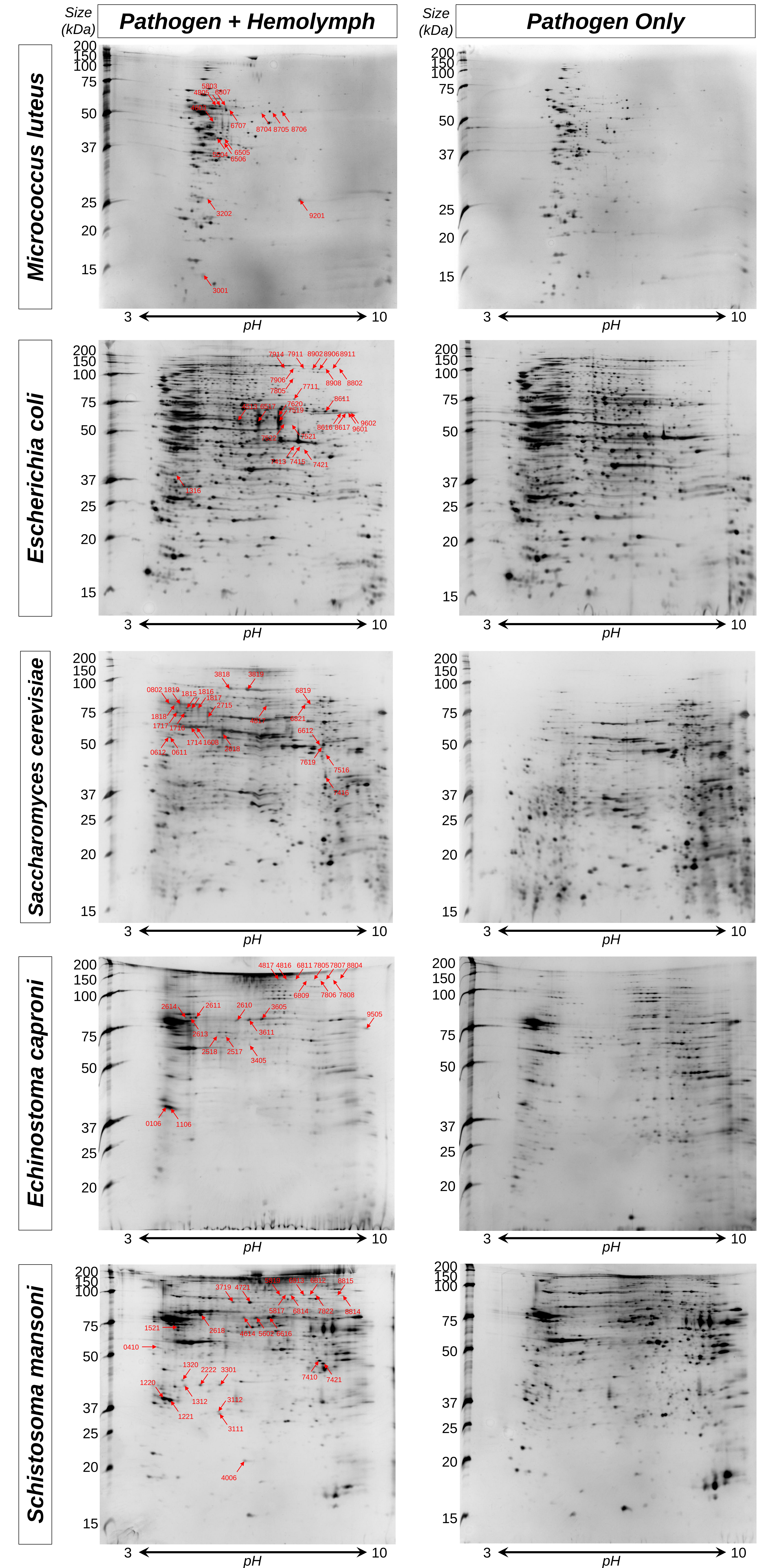

Size (kDa)
Pathogen + Hemolymph
Pathogen Only
Micrococcus luteus
Escherichia coli
Saccharomyces cerevisiae
Echinostoma caproni
Schistosoma mansoni
200
150
100
75
50
37
25
20
15
3
pH
10
5803
4805
6807
6707
8704 8705 8706
6505
6506
3202
9201
3001
7914 7911 8902 8906 8911
7906
7805
7711
8908
8802
8611
7620
7519
9602
8616 8617
9601
7521
7413 7415
7421
1316
3818
3819
0802 1819
1815 1816
1817
2715
6819
1818
6821
1717
1716
1714 1608
6612
2618
0612 0611
7619
7516
7416
4817 4816 6811 7805 7807 8804
6809
7806 7808
2614
2611
2610
3605
9505
2613
3611
2518
2517
3405
0106
1106
3719 4721
8815
5817
6814
7822
8814
1521
2618
4614 5602 6616
0410
1320
2222
3301
7410
7421
1220
1312
3112
1221
3111
4006
